# Description of the last-instar larva and pupa of a leaf-mining hispine – *Prionispa champaka* Maulik, 1919 (Coleoptera, Chrysomelidae, Cassidinae, Oncocephalini)

**DOI:** 10.3897/zookeys.729.21041

**Published:** 2018-01-16

**Authors:** Chengqing Liao, Peng Liu, Jiasheng Xu, Charles L. Staines, Xiaohua Dai

**Affiliations:** 1 Leafminer Group, School of Life and Environmental Sciences, Gannan Normal University, Ganzhou, Jiangxi 341000, China; 2 Department of Entomology, National Museum of Natural History, Smithsonian Institution, P. O. Box 37012, Washington, DC 20013-7012, USA; 3 National Navel-Orange Engineering Research Center, Ganzhou, Jiangxi 341000, China

**Keywords:** Cassidinae, chaetotaxy, immature stages, leaf-mining hispines, morphology, Oncocephalini, *Pollia*, *Prionispa*

## Abstract

The last-instar larva and pupa of *Prionispa champaka* Maulik, 1919 are described and figured in detail. The chaetotaxy of the head, mouthparts, legs, and dorsal and ventral surfaces of the body are given. The larva of *P. champaka* mine in the leaves of *Pollia japonica* Thunb. (Commelinaceae) and pupate in the base of the mid-ribs. The adults were also observed feeding on the leaves of *Pollia siamensis* (Carib.) Faden ex D. Y. Hong. The prominent diagnostic characters of immature stages of other species of the three genera of Oncocephalini (*Prionispa*, *Chaeridiona*, and *Oncocephala*) are discussed.

## Introduction

The genus *Prionispa* Chapuis, 1875 is a member of the tribe Oncocephalini, Chapuis 1875 (Chrysomelidae: Cassidinae) and consists of 29 described species occurring in the oriental tropics (Staines 2015). Seven species are recorded from China, especially in Yunnan Province: *P. champaka* Maulik, 1919, *P. cheni* Staines, 2007 (replacement name for *Chaeridiona tuberculata* Chen & Yu in Chen et al. 1986), *P. clavata* (Yu, 1992) (as *C. clavata*, Hunan), *P. dentata* Pic, 1938, *P. houjayi* Lee et al., 2009 (Taiwan), *P. opacipennis* Chen & Yu, 1962, and *P. sincia* Gressitt, 1950 (Fujian). This genus can be distinguished from the other genera in Oncocephalini, such as *Chaeridiona* Baly, 1869 and *Oncocephala* Agassiz, 1846, by the following characters: head with a distinct longitudinal ridge but without protuberance between the antennal bases; antennae not striate, third antennomere longer than the anterior two antennomeres combined; the labial palpi with three palpomeres; and the pronotum without tubercles (Chen et al. 1986).

So far only five *Prionispa* species have been recorded with host plants: *P. champaka* feeding on the leaves of an unidentified Zingiberaceae (Hua 2002), *P. dentata* feeding on several plant species of the family Zingiberaceae (Hua 2002) and Commelinaceae (Chaboo et al. 2010), *P. fulvicollis* (Guérin-Méneville, 1830) infesting *Pollia thyrsiflora* Endl. ex Hasskarl (Commelinaceae) (Taylor 1937), *P. houjayi* infesting *Disporum kawakamii* Hayata (Liliaceae) (Lee et al. 2009), and *Prionispa tuberculata* Pic, 1926 associated with *Ipomoea batatas* Poir. (Convolvulaceae) (Mo 1956). In the present study, we found the larvae of *P. champaka* mining in the leaves of *Pollia japonica* Thunb. (Commelinaceae) in Jiangxi Province, China, and we also made some biological observations.

## Materials and methods

**Figures 1–3.**
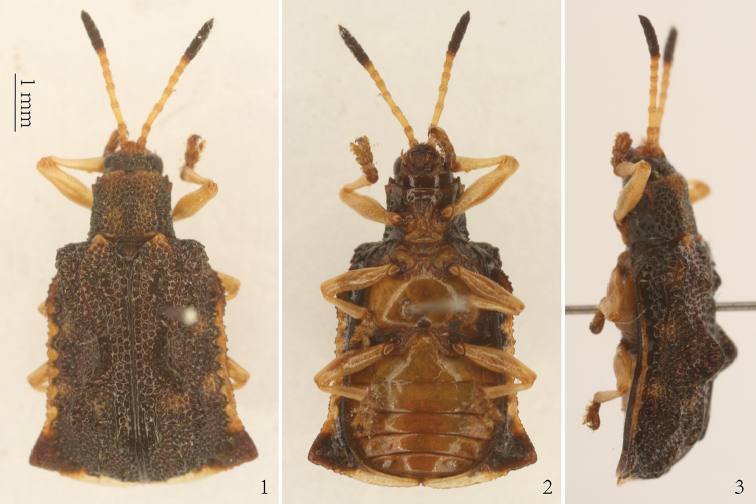
*Prionispa champaka*. **1** Dorsal view **2** Ventral view **3** Lateral view.

Immatures of *Prionispa champaka* were collected on wild plants (natural host plants) that were placed in plastic zip-lock bags. Then larvae and pupae were reared and observed in the laboratory. Field-collected and laboratory-emerged adults were preserved as pinned specimens (Figs 1–3) and identified using the keys of Chen et al. (1986). Host plants from Jiangxi Province were identified by plant experts.

All immatures were collected at Anjishan Provincial Forest Park (Longnan County, Jiangxi Prov.) from 2015 to 2017, on *Pollia japonica* Thunb. (Commelinaceae). One adult was collected at Jiulianshan National Nature Reserve (Longnan County, Jiangxi Prov.) in July 2016 (without host plant note), and one adult was collected at Bawangling National Forest Park (Changjiang County, Hainan Prov.) in August 2016, on *Pollia siamensis* with feeding channels of adults.

Three mature larvae, three pupae, and three pupal exuviae were examined morphologically. Larvae and pupae were preserved in anhydrous ethanol. For microscopic study, heads of the larvae were separated from the rest of body and then the mouthparts were dissected. The photos of adults were made using a Cannon EOS 7D camera and macro lenses; dissection of heads and mouthparts were done using a Motic SMZ-140 and Olympus SZX2-ILLT stereomicroscope; figures and examination were obtained using an Optika B-292 microscope and Cannon EOS 70D camera. Descriptions of immature stages follows Świętojańska et al. (2006). Terminology of the chaetotaxy of the head follows Borowiec and Świętojańska (2003).

All studied material (mature larvae, pupae and exuviae) and adults were deposited at the Leafminer Group, School of Life and Environmental Science, Gannan Normal University, China.

## Result

### Description of *Prionispa champaka* Maulik, 1919

**Mature larva** (Figs 4–5, 8–18)

**Figures 4–7.**
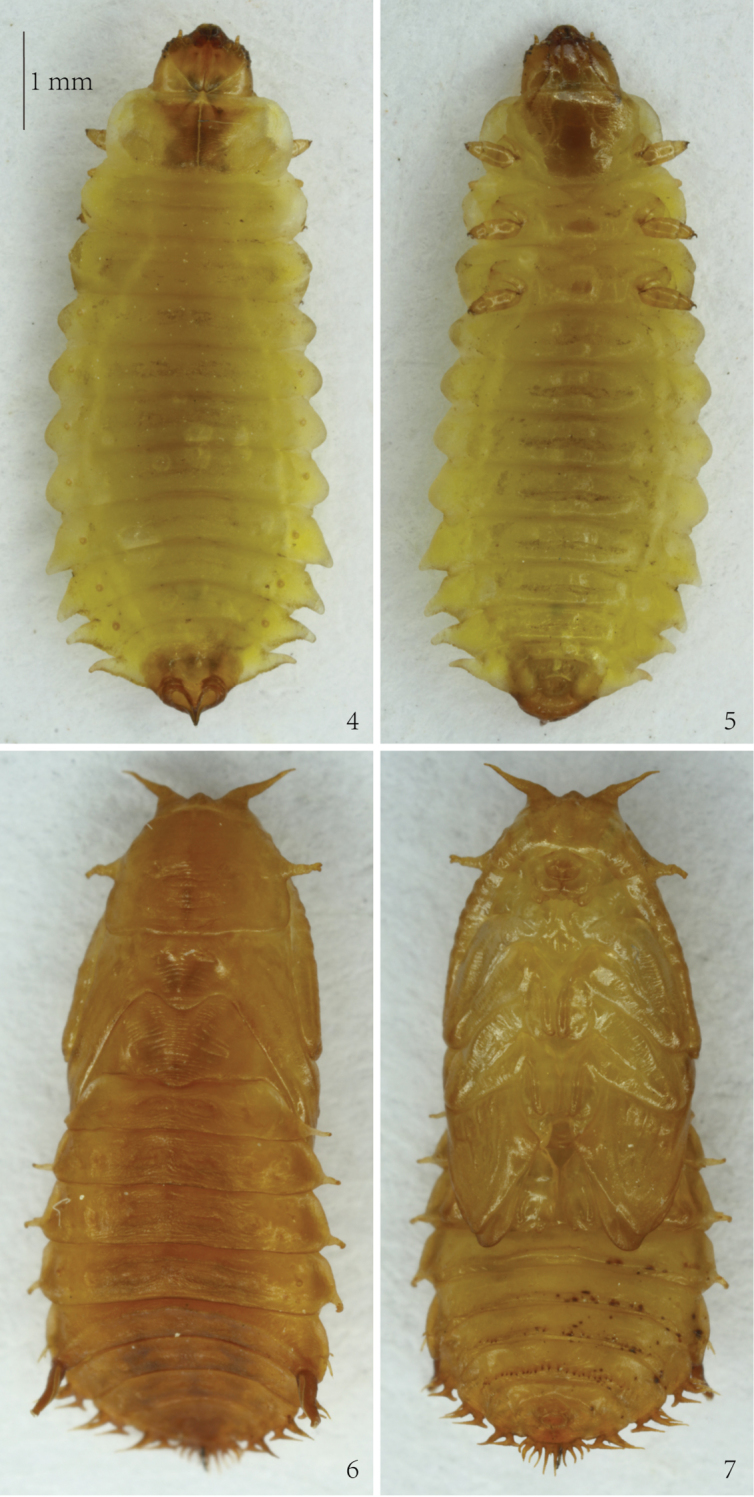
*Prionispa champaka*. **4** Larva, dorsal view **5** Larva, ventral view **6** Pupa, dorsal view **7** Pupa, ventral view.

Length of mature larva 6.5–6.6 mm without head, width of body 1.8–1.9 mm across pronotum and 2.9–3.0 mm across abdominal segment IV. Body distinctly flattened dorso-ventrally, mature larvae are widest across abdominal segments IV-V (Figs 4–5, 17–18). Body yellowish, with dark brown head, brown spiracles, and pronotum basally with two irregular brown patches and a pale longitudinal line, brown trapezoidal patch on prosternum, brown legs and dark brown claws. Body of alcohol-preserved larvae somewhat lighter in color.

*Body* with four pairs of lateral scoli placed on abdominal segments V-VIII (Figs 4–5, 17–18). Lateral scoli triangular, without lateral branches but with one seta apically and three setae at base (two placed dorsally, one ventrally). Abdominal segments I–IV without lateral scoli but with two setae laterally and one seta ventrally. Granulation of body distinct in all examined larvae. Minute setae on anterior margin of each tergite and sternite; tergites and sternites covered with short pointed setae. Tergites of meso-, metathorax and abdominal segments I–II with two transverse grooves, tergites of abdominal segments III–VII and sternites I–VIII with one transverse groove (Figs 17–18). Asperites present at the anterior margin of pronotum and around each transverse groove.

*Pronotum* on each side with 14 setae arranged in a constant pattern (Fig. 17). Meso- and metanotum with six setae on anterior margin (two pairs in the middle and a pair laterally); a row of eight setae running across segment; three setae on each side laterally and three setae placed on each lateral margin. Abdominal tergites I–VII with four minute setae on anterior margin; two rows of setae running across segment, both with four setae; two setae placed close to each spiracle. Abdominal tergite VIII with four minute setae on anterior margin; one seta placed on each side laterally; three rows of setae running across segment: anterior with four setae, next with two, and posterior with two setae placed between spiracles (close to each other). Posterior margin of spiracle VIII with eight setae on each side.

*Prosternum* with two rows of setae medially (anterior with two setae, posterior with four setae) and four setae on each side at base of leg (Fig. 18). Meso- and metasternum with two setae on anterior margin; four setae medially; and three minute setae on each side at base of leg. Abdominal sternites I–VIII with a pair of minute seta on anterior margin medially; with rows of four setae running across segment medially; and two setae on each side of sternite laterally.

Nine pairs of distinct *spiracles* (Figs 4, 17): one placed on lateral margin of mesothorax anteriorly, and eight placed on abdominal tergites. Spiracles of thorax more elevated than those on abdomen, spiracles of abdominal tergites I–VII approximate same in size, but spiracles of abdominal tergite VIII form triangular tip of body.

**Figures 8–16.**
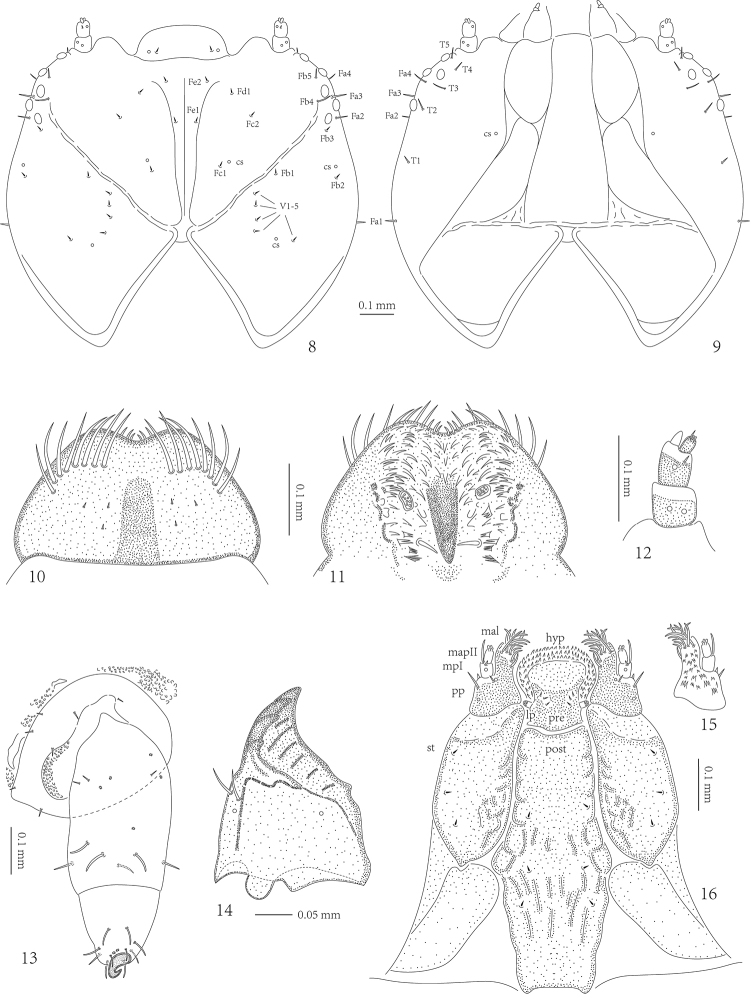
*Prionispa champaka*, larva. **8** Dorsal view of head: cs - campaniform sensilla **9** Ventral view of head. **10** Dorsal view of labrum **11** Ventral view of labrum **12** Antenna **13** Leg **14** Mandible **15** Dorsum of palpiger and maxillary palp **16** Ventral views of maxillae and labium. Abbreviations: hyp - hypopharynx; lp - labial palp; mal - mala; mpI - first segment of maxillary palp; mpII - second segment of maxillary palp; post - postmentum; pp - palpifer; pre - prementum; st - stipes.

*Head* well sclerotized, prognathous, partially retracted into pronotum (Figs 8–9). Epicranial stem absent; median endocarina wide, extending between frontal arms; frontal arms V-shaped, fronto-clypeal suture present; clypeus flat and with a pair of setae and one pair of campaniform sensilla. Frons with two short setae (Fd1 and Fe2) placed anteriorly, two short setae (Fc1 and Fc2) and one campaniform sensillum between median endocarina and frontal arm, one short seta (Fb3) and two long setae (Fb4 and Fb5) laterally close to frontal arm, one short seta on median endocarina (Fe1); vertex with seven short setae (Fb1, Fb2, and V1–5) and two campaniform sensilla (one between setae V4 and V5, one close to seta Fb2). One long seta (Fa1) placed on lateral margins close to pronotum, three long setae (Fa2, Fa3, and Fa4) close to stemmata. Temporal side with one campaniform sensillum and five setae: one shorter (T1), four longer (T2, T3, T4, and T5).

Six stemmata on each side of head (Figs 8–9). Antenna (Fig. 12) with three antennomeres, set in membranous ring; 1^st^ antennomere stout, approximately as wide as long, with two campaniform sensilla; 2^nd^ antennomere slightly slender, longer than wide, with one small seta laterally and one campaniform sensillum dorsally, prominent sensory appendix at apex close to base of 3^rd^ antennomere; 3^rd^ antennomere distinctly longer than wide, with one small seta and two peg-like sensilla at apex.

**Figure 17.**
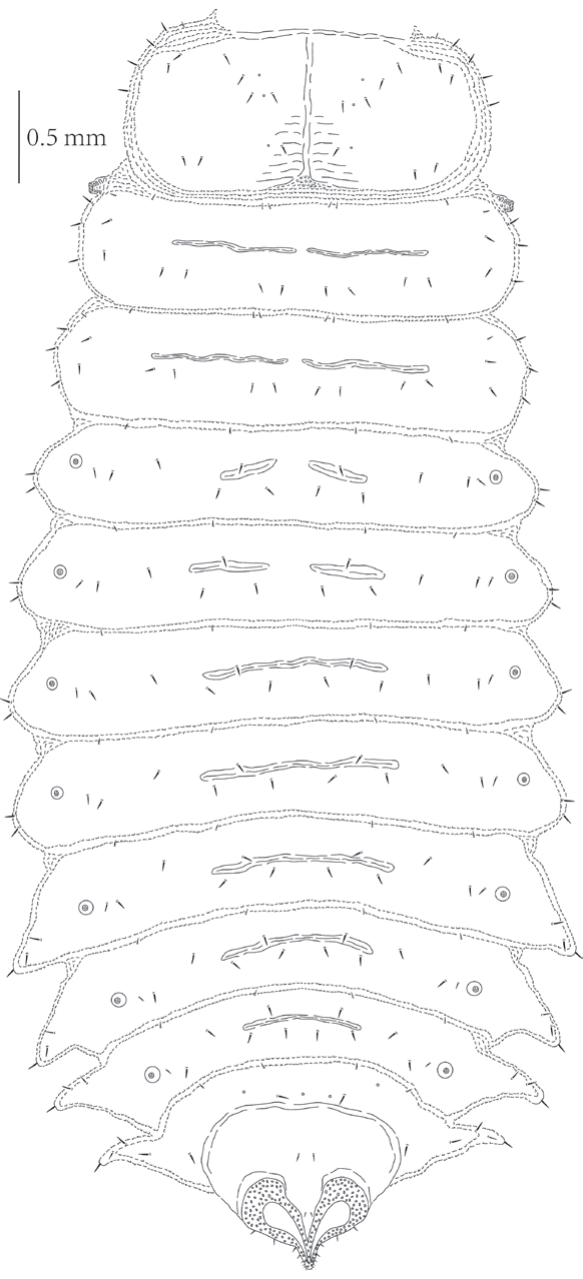
*Prionispa champaka*, last instar larva, dorsal view.

**Figure 18.**
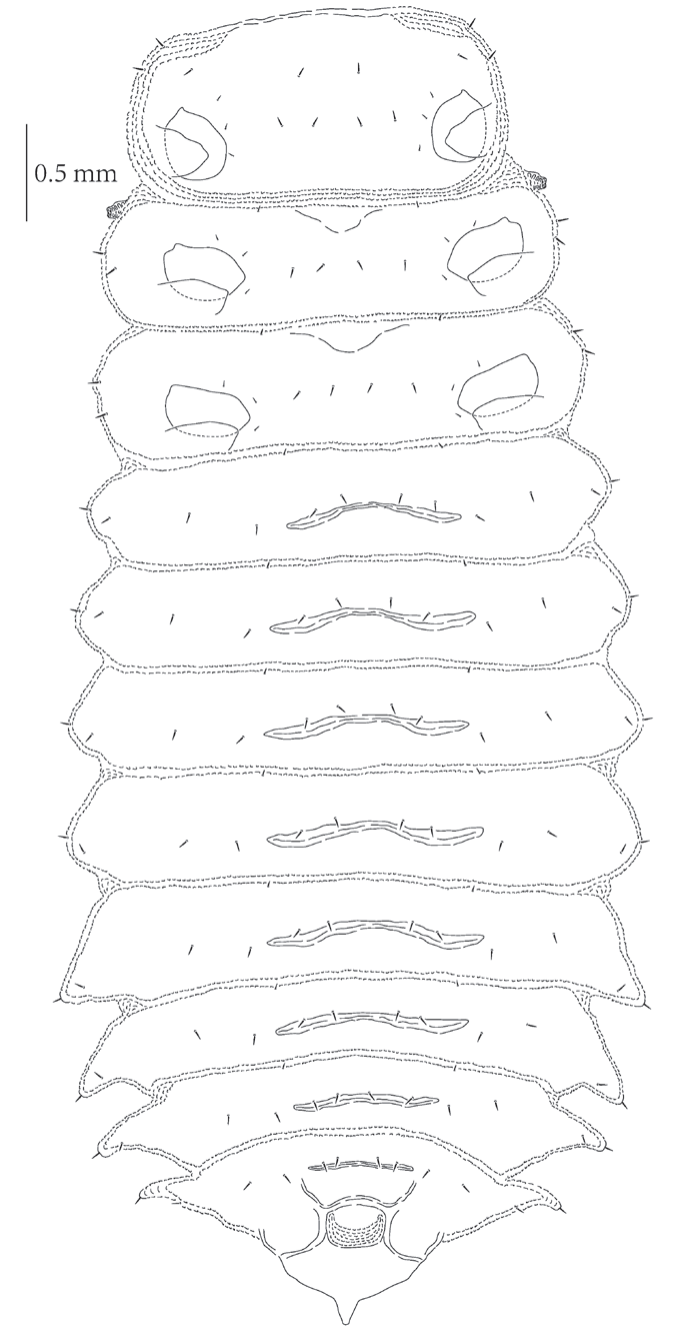
*Prionispa champaka*, last instar larva, ventral view.

Labrum approximately two times wider than long, anterior margin emarginate (Figs 10–11). Anterior margin with eight stout, long, pointed setae on each side. Dorsal surface of labrum with six short setae medially. Mid and anterior part of ventral surface (epipharyngeal area) with numerous stout spines; lateral parts with tiny spines; two irregular groups of a few small sensilla medially.

Mandibles heavily sclerotized, with four prominent teeth, followed by some tiny teeth (Fig. 14); two setae and two campaniform sensilla on dorsal side (one close to setae).

Maxillae and labium connate. Each stipes (st) with three short pointed setae laterally (Figs 15–16). Palpifer (pp) with three setae (one seta distinctly longer than others) and two campaniform sensilla ventrally, and numerous short spines dorsally. Maxillary palp (mp) with two palpomeres: first palpomere with one long seta and one short seta at apex, and one campaniform sensillum ventrally; second palpomere with a group of peg-like sensilla at apex. Mala (mal) with twelve long pointed setae apically. Hypopharynx (hyp) covered with numerous spines. Labial palp (lp) with one palpomere, with a group of small peg-like sensilla at the apex. Prementum (pre) with three setae on each side. Postmentum (post) with three short setae placed on each side medially.

*Legs* stout, consist of three segments: coxa, femur, and tibiotarsus (Fig. 13). Tibiotarsus armed apically with heavily sclerotized, short, curved, single, simple claw. Coxa with four setae placed along base on internal surface, three setae placed dorsally. Femur with three short pointed setae and four campaniform sensilla placed in basal half, six long pointed setae and one campaniform sensillum placed around apical half. Tibiotarsus with nine long pointed setae and two campaniform sensilla: six setae around claw, three setae, and two sensilla above claw.

**Figures 19–20.**
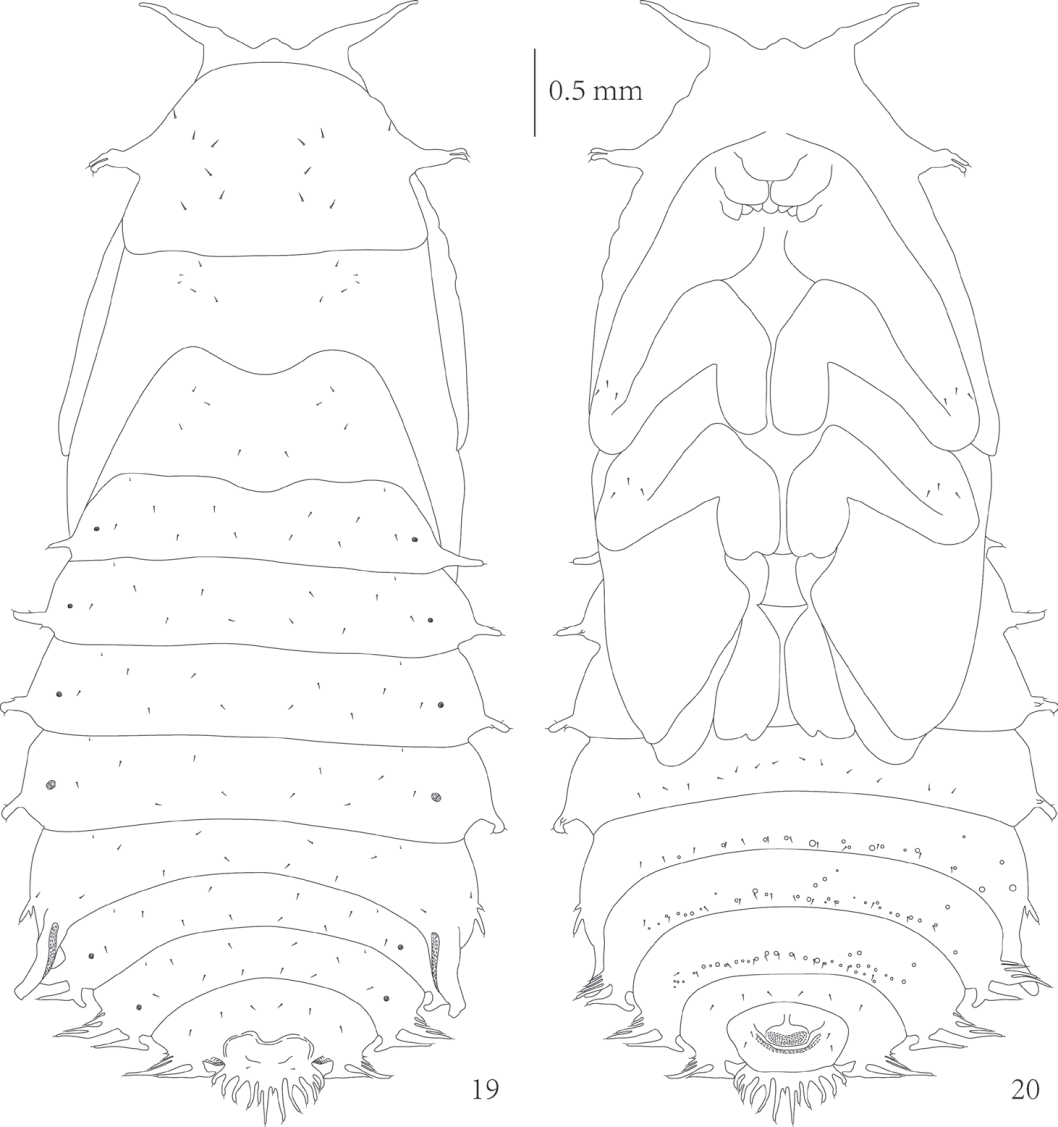
*Prionispa champaka*, pupa. **19** Dorsal view **20** Ventral view.

**Pupa** (Figs 6–7, 19–20)

Length of pupa 6.5 mm, width of body 2.0 mm across the base of pronotum and 3.0 mm across abdominal segment IV without lateral scoli. Body flattened dorso-ventrally, elongate-oval, color when alive as well as alcohol preserved pupa yellowish-brown (Figs 6–7).

*Head* with two distinct long and one short triangular processes on anterior margin (Figs 6–7, 19–20). Prothorax with a pair of two-branched lateral scoli, but meso- and metathorax without lateral scoli. Each short branch apically armed with pointed seta. Abdominal segments I–IV with single scolus on each side, these scoli basally with two to three very small tubercle-like branches. Abdominal segment V with one two-branched scolus laterally. Abdominal segments VI–VIII with five lateral scoli, usually the third and fourth scolus with a broad common stem. Each scolus or branch apically with one seta. Posterior lateral scolus of segments VI–VIII with lateral branch directed posteriorly without any seta. Segment VIII additionally with 14 (rarely 13 or 15) flattened long processes (more or less one-two processes shorter than others) placed at posterior border. Each process armed with single seta, regardless of their size or length.

*Pronotum* with one pair of setae laterally and five setae on each side medially (Fig. 19). Mesonotum with five setae on each side medially (lateral two minute setae). Metanotum with a group of three setae on each side laterally and two setae medially. *Abdominal tergites* I–VII with two minute setae at anterior border; two rows of setae running across segment, both with four setae; one seta placed close to each spiracle. Abdominal tergite VIII with two rows of setae running across segment: anterior with six setae, and posterior with two setae (placed between spiracles).

In ventral view (Fig. 20): head and mouthparts without setae; each femur of legs with three setae placed at apex (hind legs were covered by the wings, invisible); visible abdominal sternites IV–VII with rows of twelve setae running across segment posteriorly; abdominal sternite VIII with rows of six setae running across segment anteriorly, and two setae placed on each side of anus.

Abdominal segments each with a pair of *spiracles* (Fig. 19). Spiracles of segments I–III and VI–VII similar in size. Spiracles of segment IV similar and approximately two times larger than others. Spiracles of segment VIII not elevated, and similar to spiracles of segment IV. Spiracles of segment V most prominent, elongated into long appendage (respiratory horns) with elongate-oval spiracular opening, directed posteriorly. Abdominal sternites V–VII each with one row of tubercles placed posteriorly (tubercles of sternite VII are the most developed), some of them very close to setae of each sternite.

### Habitat and biological notes

**Figures 21–28.**
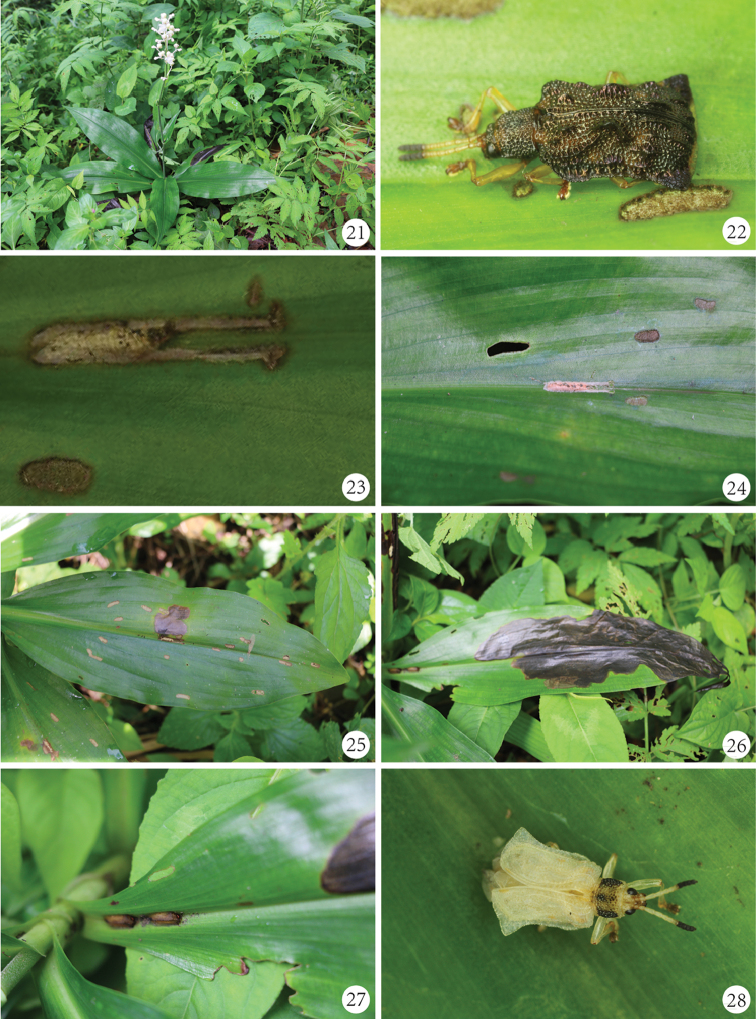
Life stages of *Prionispa champaka*. **21** *Pollia japonica*, the host plant for *P. champaka* **22** Adult of *P. champaka* and its feeding channel **23-24** An egg sheath of *P. champaka* which laid and located on the mid-rib of upper surface of a leaf **25** A leaf with new mine of hatching larvae of *P. champaka* and some linear feeding channels of adults **26** Large larval mine of mature larvae **27** Two pupal mines of *P. champaka* located at the base of petiole on the upper surface **28** Freshly emerged adult.

There is little biological information known on *P. champaka*. It was reported to feed an unidentified host plant of the family Zingiberaceae from China (Hua 2002), but without any descriptions on larval mines and adult feeding patterns. At Anjishan Provincial Forest Park from 2015 to 2017, we collected eggs, larvae, pupae and adults of this species on the leaves of *Pollia japonica* from June to July, and only mature larvae, pupae and adults in August (Figs 21–27). Additionally, we collected only one adult in 31th October 2016. At Jiulianshan National Nature Reserve (Longnan County, Jiangxi Prov.), we collected only adults from 13–19th July 2016. At Bawangling National Forest Park (Changjiang County, Hainan Prov.), we collected only adults associated with *Pollia siamensis* and its feeding channels from 4–11th August 2016. Chen et al. (1986) recorded adults of this species occurring in Yunnan Province from May to June. We consider the life cycle of *P. champaka* to be univoltine based on the information above.

The female of *P. champaka* bites a narrow line across the mid-rib of the lower canopy leaves on the plant, and then extends it along each side of the mid-rib in the same direction. The biting channel results in two short vertical lines and forms an elongate “U” shape. Subsequently, the female lays a single long egg sheath (usually comprising 5–8 eggs) at the base of the biting channel. Finally, the female covers this portion of the biting channel with feces (Figs 23–24). This shows the biting channel of the female as well as her secretion helps to protect the egg sheath. The length of the egg sheath is approximately 7–10 mm (average 8.5 mm, ten sheathes were measured), which is usually shorter than that of the female biting channel. The larvae are gregarious, usually developing in a common mine (Fig. 25). Freshly hatched larvae bore into the mesophyll of the upper leaf surface (Fig. 25). The larval mine is very broad and irregular in shape, even extending to the entire leaf (Fig. 26). The larvae deposit their feces in the mine. The mature larva leaves its original mine and builds a new one on the base of petiole (Fig. 27), in which it transforms into the pupa and then emerges as an adult. The pupal mine is an elongate channel with a distinct opening which closed by apex of pupal abdomen (Fig. 27). The freshly emerged adults are mostly white, with eyes and apical four antennomeres black, pronotum yellowish with three black longitudinal marks or completely black, and brownish tarsi (Fig. 28). Hours later, the body of adults becomes darker and harder (Fig. 22). Subsequently, the adults start to feed. The feeding channels are elongate-oval or linear striped, usually on the upper surface of leaves (Fig. 25).

## Discussion

Immature stages of only four species in the tribe Oncocephalini have been described in detail. Świętojańska et al. (2006) described and compared the mature larvae and pupae of *C. picea* Baly, 1869 and *O. quadrilobata* Guérin-Méneville, 1844. In this study, the authors indicated that the typical morphology of Old World leaf-mining hispines are a flattened body and at least abdominal segments with lateral scoli in the larval stage, and very long spiracles on the fifth abdominal segment in the pupal stage. Świętojańska and Kovac (2007) described the mature larva and pupa of *C. thailandica* Kimoto, 1998 with detailed habitat and biological notes. Lee et al. (2009) described the immature stages and adult of *P. houjayi*. Our study showed that the diagnostic characters are the lateral scoli of the thorax, the abdominal apex of the larva, and the processes on the head and pronotum of the pupa.

In the present study, a prominent diagnostic character of the larva of *P. champaka* was found: the lateral scoli on abdominal segments V–VIII. However, the lateral scoli of *P. houjayi* are present on all abdominal segments, as well as the meso- and metathorax (Lee et al. 2009). Presence of the lateral scoli on abdominal segments is variable between genera or within a genus, but at least some abdominal segments have lateral scoli, especially the posterior segments. The shape of abdominal apex is most similar to the tapering type of *C. thailandica* and *C. picea* but not the quadrate type of *P. houjayi*, indicating that this is not a constant character within the genus *Prionispa*. Furthermore, the larva of *P. champaka* has prominent labial palpi, highlighted as a unique character for *P. houjayi* (Lee et al. 2009). However, the labial palpi of *P. champaka* seem to be less prominent than those of *P. houjayi*.

The pupa of *P. champaka* is most similar to that of *P. houjayi*. These two species have a pair of prominent processes on the head and pronotum which is probably a constant character within the genus. The lateral scoli of the abdominal segments and the fifth abdominal spiracles also look very similar, but the opening of the fifth abdominal spiracle extends to the base of the spiracle. The most prominent difference from other species of these three genera is that the abdominal apex of *P. champaka* has 14 long flattened processes, whereas *P. houjayi* has only two wide flattened processes, *O. quadrilobata* has four flattened processes, and *C. thailandica* and *C. picea* respectively have two and three slender processes (Lee et al. 2009; Świętojańska et al. 2006; Świętojańska and Kovac 2007).

It can be seen from above that the lateral scoli and abdominal apex of larva and pupa are the most important diagnostic characters between different species in these three genera as well as other Old World genera of leaf-mining hispines, such as *Dactylispa* Weise, 1897 (Chen et al. 1986; Kimoto and Takizawa 1994; Lee and Cheng 2007, 2010; Zaitsev 2012; Dai et al. 2012), *Platypria* Guérin-Méneville, 1840 (Kimoto and Takizawa 1994; Liao et al. 2014), *Dicladispa* Gestro, 1897 (Chen et al. 1986; Lee et al. 2010; Świętojańska et al. 2014).

The habits of immature stages of *P. champaka* are similar to other leaf-mining chrysomelids especially hispines. The mature larvae move to another location and build a new mine (called “pupal mine” or “pupal chamber”) for pupation (Hering 1951), the same as *P. houjayi* Lee et al. (on the lower surface of the leaf close to the mid-rib) (Lee et al. 2009), *O. promontorii* Péringuey, 1898 (on the lower surface away from the leaf base) (Chaboo et al. 2010), *C. thailandica* Kimoto (on the mid-rib on the upper surface of the leaf) (Świętojańska and Kovac 2007), *Platypria melli* Uhmann, 1954 (on a vein of the leaf especially the mid-rib on the upper surface) (Liao et al. 2014), *Notosacantha vicaria* (Spaeth, 1913) (on the mid-rib on the upper surface of the leaf) (Rane et al. 2000), and some species of genus *Dactylispa* (unpublished data). The pupa of *P. champaka* can close its pupal mine with the broadened and flat caudal end of the abdomen, as can *C. thailandica* (Świętojańska and Kovac 2007).
